# Maxillary Glandular Odontogenic Cyst - report of 3 cases and literature review

**DOI:** 10.4317/jced.62938

**Published:** 2025-08-01

**Authors:** Bruno Teixeira Gonçalves Rodrigues, Estephani Martins Barcellos de Carvalho, Francisco Lopes Leal Gonçalves, Thuany Servare de Lima, Nathália de Almeida Freire, Henrique Martins da Silveira, Mônica Simões Israel, Marcele Cruz da Silva, Paulo José d’Albuquerque Medeiros, Fábio Ramoa Pires

**Affiliations:** 1 DDS, OMFS. Oral & Maxillofacial Surgery, Pedro Ernesto University Hospital, Rio de Janeiro State University, Rio de Janeiro, Brazil. Oral Medicine, Department of Diagnosis and Therapeutics, School of Dentistry, Rio de Janeiro State University, Rio de Janeiro, Brazil; 2 DDS. Oral & Maxillofacial Surgery, Pedro Ernesto University Hospital, Rio de Janeiro State University, Rio de Janeiro, Brazil.; 3 DDS. Post-graduation in Oral Medicine, São Leopoldo Mandic School, Rio de Janeiro, Brazil; 4 DDS, PhD. Oral Medicine, Department of Diagnosis and Therapeutics, School of Dentistry, Rio de Janeiro State University, Rio de Janeiro, Brazil. Post-graduation in Oral Medicine, São Leopoldo Mandic School, Rio de Janeiro, Brazil; 5 DDS, PhD. Oral & Maxillofacial Surgery, Pedro Ernesto University Hospital, Rio de Janeiro State University, Rio de Janeiro, Brazil; 6 DDS, MsC. Oral & Maxillofacial Surgery, Pedro Ernesto University Hospital, Rio de Janeiro State University, Rio de Janeiro, Brazil; 7 DDS, PhD. Oral Pathology, Department of Diagnosis and Therapeutics, School of Dentistry, Rio de Janeiro State University, Rio de Janeiro, Brazil

## Abstract

**Background:**

Glandular Odontogenic Cyst (GOC) is a rare benign lesion with unique histological features, and more aggressive growth pattern compared to other odontogenic cysts. GOC typically presents as an asymptomatic, slow-growing swelling, predominantly affecting middle-aged males and often localized in the anterior mandible. This report details three cases of GOC affecting the maxilla.

**Case Report:**

Case 1 presented a GOC mimicking a residual cyst in the maxilla. Case 2 involved a GOC located within the maxillary sinus. Case 3 showed a multilocular radiolucent GOC in the anterior maxilla. All cases were treated by surgery, and the diagnosis was confirmed through microscopic examination.

**Conclusions:**

Clinicians should consider GOC when evaluating unilocular or multilocular lesions in the maxilla.

** Key words:**Odontogenic cyst, Jaws, Maxilla, Glandular odontogenic cyst.

## Introduction

Glandular Odontogenic Cyst (GOC) is a rare, benign condition with unique histological features, showing a more aggressive growth pattern in comparison to other odontogenic cysts (OCs). GOC accounts for approximately 0.4% of all OCs [1], and tends to occur predominantly in the anterior mandible [1,2]. In rare cases it may affect the maxilla [1,2].

GOC typically manifest as an asymptomatic, slow-growing swelling, most commonly affecting middle-aged males. Symptoms such as pain and facial edema may eventually arise from secondary infections. GOC usually presents as a well-defined unilocular radiolucency with sclerotic borders. However, a multilocular pattern can be also observed, indicating a potentially more aggressive behavior that may cause significant local osteolysis [1-3].

Histological examination is essential for diagnosing GOC. It typically features a cystic capsule of fibrous connective tissue lined by non-keratinized epithelium with a superficial layer composed of columnar or cuboidal cells, often exhibiting cilia and goblet cells. Additional histological features such as glandular or pseudoglandular structures, along with microcyst formation, are also common. Histological analysis is crucial for differentiating GOC from other entities, including periapical cysts, odontogenic keratocysts, ameloblastomas and mucoepidermoid carcinomas [1-5].

Treatment for GOC is surgical intervention. Enucleation with curettage is the most common approach, and carries a recurrence rate of approximately 21% [6]. Alternatively, marsupialization before surgery is recommended in some cases to reduce the extent of the procedure and to avoid damage to important anatomical structures. However, due to the aggressive nature of GOC, some authors suggest that radical surgery through resection should be considered [1-6].

In this report, we present a case series of GOC affecting the maxilla, highlighting their clinical, radiographic, and microscopic features.

Case Report

- Case 1 – GOC mimicking a residual cyst

**Figure 1:**
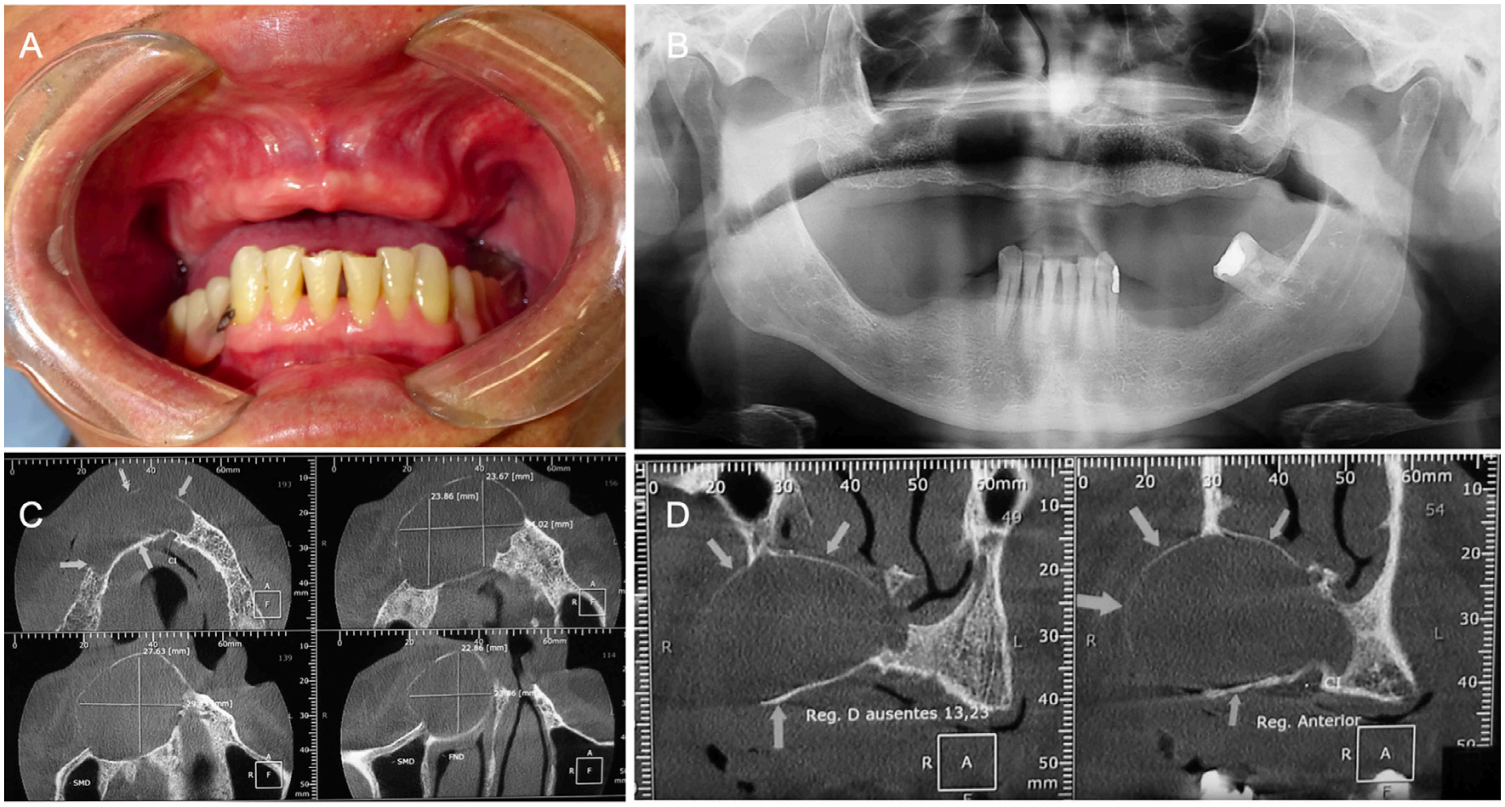
Case 1 clinical and radiographic features. A, Intraoral examination showed a maxillary swelling covered by normal oral mucosa. B, A well-defined unilocular radiolucent image could be observed on panoramic radiograph, mimicking a residual cyst. C-D, CBCT features demonstrating a hypodense lesion causing maxillary cortical expansion without perforation.

A 68-year-old male was referred for evaluation of a radiolucent lesion in the anterior maxilla, detected on panoramic radiograph. Patient’s medical history was unremarkable, and he denied tobacco and alcohol use. No clinical alterations were noted on extraoral exam; however, a slight maxillary swelling could be observed on intraoral examination (Fig. 1A). Orthopantomograph revealed a well-defined, unilocular radiolucency in the right anterior maxilla, extending slightly across the midline (Fig. 1B). Cone-beam computed tomograph (CBCT) scans showed a large hypodense image causing cortical expansion (Fig. 1C-D). Clinical diagnosis included residual cyst and odontogenic keratocyst. An incisional biopsy was performed under local anesthesia and the specimen was sent to microscopic analysis. Histological examination revealed a cyst cavity lined by non-keratinized stratified squamous epithelium, with cuboidal cells in the superficial layer and small areas of microcyst formation, thus establishing the diagnosis of GOC. The patient was referred to a maxillofacial surgeon and enucleation of the lesion was performed under general anesthesia. Histological analysis of the surgical specimen confirmed the diagnosis of GOC. Patient was lost to follow-up.

- Case 2 – GOC in the right maxillary sinus

**Figure 2:**
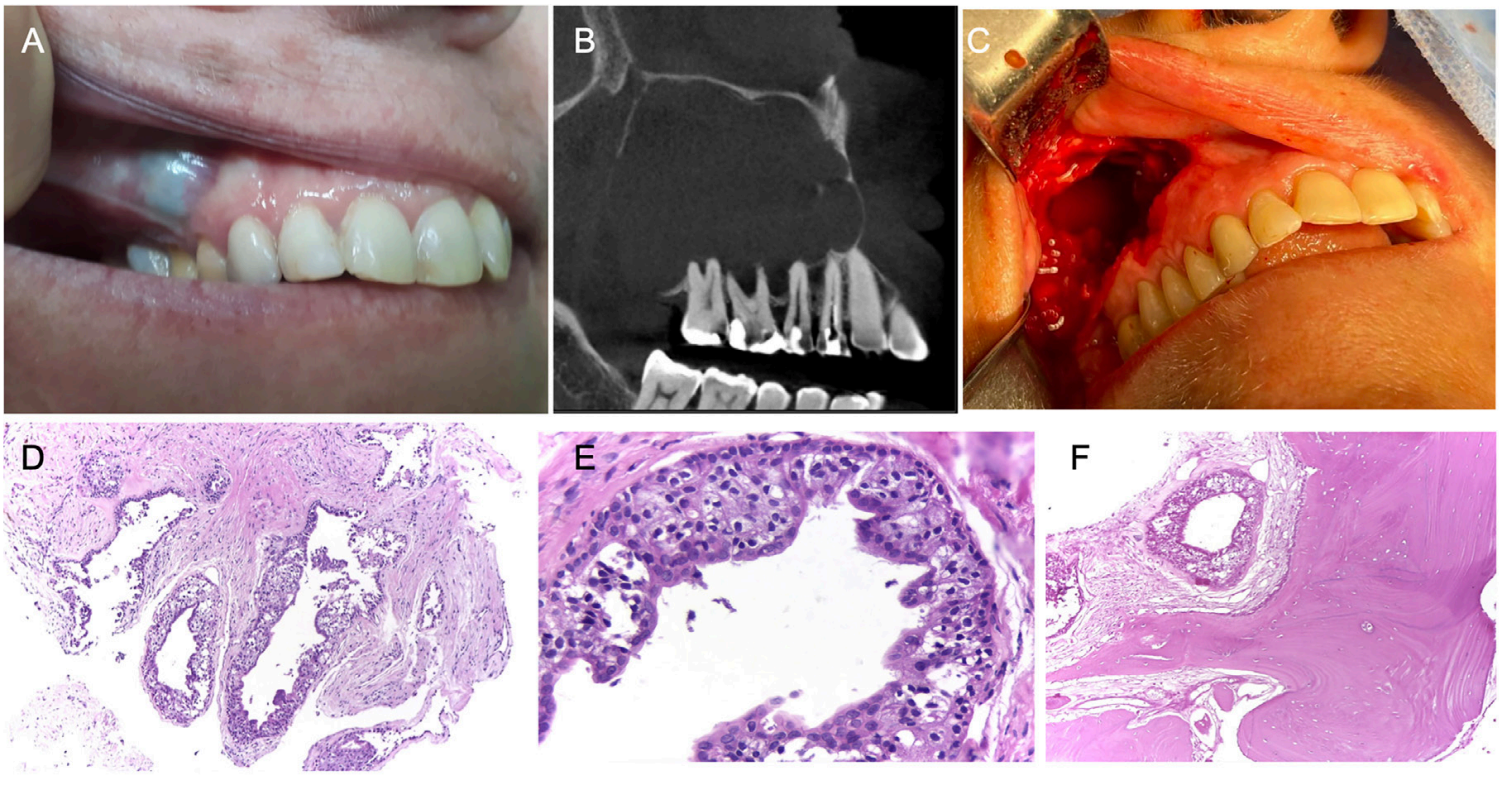
Case 2 featured a GOC at the posterior maxilla. A, intraoral examination showed a bluish swelling located in the right maxillary posterior region. B, CBCT features showing an extensive multilocular hypodense lesion within the maxillary sinus, perforating the anterior maxillary wall and extending superiorly to the orbital floor. C, Surgical aspect of the large cystic cavity. D-E, Histological features of GOC - cystic cavity lined by non-keratinized epithelium with the superficial layer composed by columnar or cuboidal cells presenting cilia and goblet cells, pseudoglandular structures and microcyst formation (A – HE, original magnification - 100x; B – HE, original magnification - 400x). F, cystic structures infiltrating adjacent bone tissue (HE, original magnification - 100x).

A 56-year-old woman was referred for evaluation of a 2-month-lasting oral swelling in the right posterior maxillary alveolar mucosa. Patient also complained of ipsilateral ophthalmic pain and facial swelling. Patient’s medical history was unremarkable, and she denied tobacco and alcohol use. Extraoral examination revealed a slight facial swelling. Intraoral examination showed an asymptomatic bluish tense swelling with intact smooth surface mucosa, located in the right upper alveolar mucosa, between maxillary molars (#16 and #17) and premolars (#14 and #15) (Fig. 2A). All teeth were vital. CBCT scans revealed a large multilocular hypodense image causing bone cortical resorption, located in the right posterior maxilla within the maxillary sinus (Fig. 2B). Clinical differential diagnosis included odontogenic keratocyst and ameloblastoma. An incisional biopsy with marsupialization was performed under local anesthesia. Histological analysis established the diagnosis of GOC. After eight months, enucleation with curettage of the lesion was performed under general anesthesia, and analysis of the surgical specimen confirmed the diagnosis of GOC (Fig. 2C-F). Patient’s recovery was uneventful, and no signs of recurrence were observed one year after the surgical procedure.

- Case 3 – GOC as an extensive multilocular lesion in the anterior maxilla

**Figure 3:**
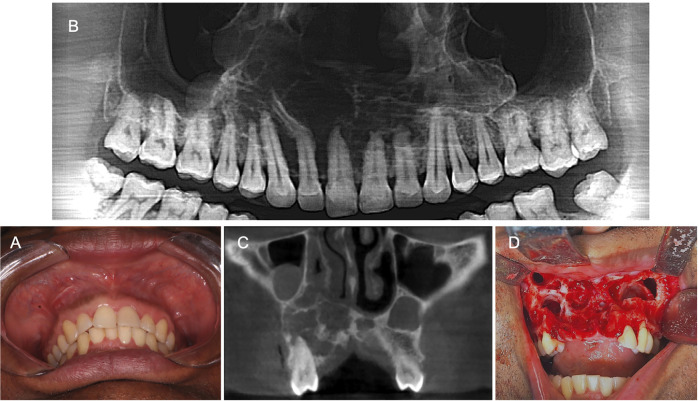
Case 3 clinical and tomographic findings. A, Intraoral examination showed no alterations. B, CBCT panoramic reconstruction demonstrating a multilocular image with sclerotic borders located in the anterior maxilla. C, CBCT scan showing teeth roots associated with the lesion and rupture of the lower cortical of the nasal cavity. D, After performing a total mucoperiosteal flap and removing the teeth in association with the lesion, observe the presence of multiple cystic spaces within the bone.

A 44-year-old male was referred for evaluation of a maxillary lesion detected on routine radiographic examination. Patient’s medical history was non-contributory, and he denied tobacco and alcohol use. No abnormalities were found on both extraoral and intraoral clinical examination (Fig. 3A). CBCT scans revealed an ill-defined multilocular hypodense image extending from the upper right first molar (#16) to the upper left first molar (#26), crossing the midline (Fig. 3B-C). Patient had been previously submitted to an incisional biopsy with a diagnosis consistent with GOC. Enucleation with curettage was performed under general anesthesia. Extraction of all maxillary incisors and the right canine was also required due to the association of the cyst with the teeth roots (Fig. 3D). Histological examination of the surgical specimen confirmed the diagnosis of GOC. Patient’s recovery was uneventful, and no signs of recurrence were observed in the latest follow-up.

 Table 1 summarizes the clinical and radiological features from each case.

## Discussion

First described by Padayachee and Van Wyk [7] in 1987 as “sialo-odontogenic cyst,” the nomenclature GOC was formally suggested one year later by Gardner *et al*. [8]. The recent World Health Organization classification update of head and neck tumors [9] defines GOC as a developmental cyst with epithelial features that mimic salivary gland or glandular differentiation. GOC typically affects the mandible rather than the maxilla, with a ratio of 2.73:1, most commonly in the anterior region, often crossing the midline [1-9]. There is a slight male predilection (1.15:1 male to female ratio), with the condition typically affecting individuals within the fifth and sixth decades of life, with a mean age of 48 years [6]. GOC typically presents as a slow-growing, painless swelling [1-7], as observed in case 1. Radiologically, a well-defined unilocular radiolucent lesion is more common than a multilocular pattern [10]. The three cases reported in this study align with the literature, as all patients were middle-aged, and men were affected twice as frequently. However, GOC occurring in the maxilla is rare, especially in the posterior region, as observed in case 2.

A recent multicentric study by Nel *et al*. [5], analyzing 92 GOC, found that only 29 (32%) were located in the maxilla. Notably, 2 patients (7%) presented with the cyst in the posterior maxillary region, and most lesions appeared as unilocular radiolucencies. Similarly, a case series reported by Lonni *et al*. [4] also identified the maxilla as an uncommon affected site, with all lesions being unilocular and being located in the anterior region. Additionally, a retrospective study by Heiliczer *et al*. [3] reported that 40% of the cases occurred in the maxilla. Therefore, the present case series contributes to the existing literature by documenting three new cases of maxillary GOC with uncommon features — two presenting with a multilocular pattern and one located in the posterior region.

Diagnosis of GOC can be challenging, as its features overlap with other conditions, such as radicular/residual cysts, odontogenic keratocysts, ameloblastomas and mucoepidermoid carcinoma [10-12]. In case 1, clinical diagnosis was residual cyst, due to its presentation as a well-defined unilocular radiolucency at a site of previous tooth extraction. In case 2, clinical diagnosis included ameloblastoma, due to the local swelling produced by the lesion and the multilocular radiolucent image. Microscopic examination is essential to confirm diagnosis, and to exclude other potentially clinical, radiological and histological mimickers, especially ameloblastomas and mucoepidermoid carcinoma. This is crucial to define treatment planning and prognostic impact.

Treatment for GOC typically involves surgical procedures, such as enucleation with curettage, marsupialization/decompression prior to enucleation/curettage, or marginal resection [1-15]. GOC has a higher recurrence rate compared to other benign odontogenic cysts, with an approximate rate of 21.6% [6]. While many authors suggest that conservative treatment leads to a higher recurrence rate, a review of 169 cases by Chrcannovic *et al*. [6] demonstrated no statistical difference between enucleation and resection, ultimately favoring enucleation/curettage for better recovery and rehabilitation. In this study, all three cases were treated with enucleation/curettage. However, case 2 involved a large, painful GOC within the maxillary sinus, likely causing discomfort due to compression of neurovascular structures. To relieve the symptoms and minimize the surgical impact, marsupialization was performed. Case 3 involved a multilocular GOC in the anterior maxilla, with the cyst in intimate contact with the roots of the upper incisors and right maxillary canine, necessitating the extraction of these teeth.

GOC is a benign developmental cyst that is uncommon in the maxilla. Its clinical and radiographic features closely resemble other jaw cysts and tumors. Therefore, microscopic analysis is essential to achieve an accurate diagnosis and determine the appropriate treatment. This case series highlighted the importance of including GOC in the differential diagnosis of maxillary radiolucencies. However, due to the limited sample size, further studies are needed to better understand behavior of maxillary GOC.

## Figures and Tables

**Table 1:** Table Summarizes the clinical and radiological features from each case.

Case	1	2	3
Age	68	56	44
Sex	Male	Female	Male
Signs/symptons	Radiographic finding	Oftalmic pain; intraoral volumen growth	Radiographic finding
Location	Anterior maxillary region	Posterior maxillary region	Anterior maxillary region
Radiographic aspect	Unilocular radiolucency	Multilocular radiolucency	Multilocular radiolucency
Treatment	Enucleation	Marsupialization before enucleation	Enucleation + teeth extraction

## Data Availability

The datasets used and/or analyzed during the current study are available from the corresponding author.
